# Could international compulsory licensing reconcile tiered pricing of pharmaceuticals with the right to health?

**DOI:** 10.1186/s12914-014-0037-4

**Published:** 2014-12-18

**Authors:** Gorik Ooms, Lisa Forman, Owain D Williams, Peter S Hill

**Affiliations:** Department of Public Health, Institute of Tropical Medicine, Nationalestraat 155, 2000 Antwerp, Belgium; Law and Development Research Group, Faculty of Law, University of Antwerp, Antwerp, Belgium; Dalla Lana School of Public Health, University of Toronto, Toronto, Canada; Munk School of Global Affairs, University of Toronto, 155 College Street, Toronto, M5T 3M7 Ontario Canada; School of Population Health, The University of Queensland, Herston Road, Brisbane, 4006 Australia

**Keywords:** Right to health, Tiered pricing, TRIPS, Medicines, Patent, Compulsory licensing

## Abstract

**Background:**

The heads of the Global Fund and the GAVI Alliance have recently promoted the idea of an international tiered pricing framework for medicines, despite objections from civil society groups who fear that this would reduce the leeway for compulsory licenses and generic competition. This paper explores the extent to which an international tiered pricing framework and the present leeway for compulsory licensing can be reconciled, using the perspective of the right to health as defined in international human rights law.

**Discussion:**

We explore the practical feasibility of an international tiered pricing and compulsory licensing framework governed by the World Health Organization. We use two simple benchmarks to compare the relative affordability of medicines for governments – average income and burden of disease – to illustrate how voluntary tiered pricing practice fails to make medicines affordable enough for low and middle income countries (if compared with the financial burden of the same medicines for high income countries), and when and where international compulsory licenses should be issued in order to allow governments to comply with their obligations to realize the right to health.

**Summary:**

An international tiered pricing and compulsory licensing framework based on average income and burden of disease could ease the tension between governments’ human rights obligation to provide medicines and governments’ trade obligation to comply with the Agreement on Trade-Related Aspects of Intellectual Property Rights.

## Background

While the heads of the Global Fund and GAVI Alliance promote the idea of a global tiered pricing framework [1], civil society groups are asking them to abandon and dissolve the taskforce that was created to explore and develop this idea – originally named the ‘Blue Ribbon Taskforce’ [2], recently renamed the ‘Equitable Access Initiative’ [3]. One of these civil society groups is Oxfam, which until recently was a proponent of an international tiered pricing framework [4]. Oxfam’s position shift may be due to the fact that in the international tiered pricing framework it had in mind, the pricing of patent-protected medicines would be set by “an international public health body such as the WHO” [4], while the Equitable Access Initiative seems to rely ultimately on voluntary discounts by patent-owning companies. For example, Seth Berkley, the CEO of the GAVI Alliance, writes: “So although it is for manufacturers to set the prices of vaccines, the tiers would act as a guide irrespective of whether they are multinational corporations or developing country vaccine manufacturers” [1].

**Table 1:** **Reasonable and real discounts for Atripla**

**Country name**	**GNI discount**	**HIV prevalence discount**	**Combined discount**	**Merck discount**
Congo, Dem. Rep.	99.486	77.273	99.883	95
Burundi	99.463	80.769	99.897	95
Malawi	99.284	97.685	99.983	95
Liberia	99.173	72.222	99.770	95
Ethiopia	99.150	80.769	99.837	95
Niger	99.128	50.000	99.564	95
Madagascar	99.038	50.000	99.519	95
Guinea	99.016	85.294	99.855	95
Eritrea	98.994	64.286	99.641	95
Uganda	98.927	96.528	99.963	95
Togo	98.882	91.379	99.904	95
Central African Republic	98.860	0.000	98.860	95
Gambia, The	98.860	80.769	99.781	95
Guinea-Bissau	98.860	93.590	99.927	95
Mozambique	98.860	97.748	99.974	95
Tanzania	98.725	95.098	99.938	95
Sierra Leone	98.703	83.333	99.784	95
Rwanda	98.658	91.379	99.884	95
Zimbabwe	98.547	98.299	99.975	95
Mali	98.524	72.222	99.590	95
Burkina Faso	98.502	75.000	99.625	95
Afghanistan	98.479	0.000	98.479	95
Nepal	98.435	16.667	98.696	95
Benin	98.323	77.273	99.619	95
Haiti	98.301	88.095	99.798	95
Chad	98.278	90.741	99.841	95
South Sudan	98.233	90.741	99.836	?
Bangladesh	98.122	0.000	98.122	95
Comoros	98.122	88.095	99.776	95
Kenya	98.077	95.902	99.921	95
Cambodia	98.032	68.750	99.385	95
Tajikistan	98.032	16.667	98.360	92
Kyrgyz Republic	97.786	16.667	98.155	92
Senegal	97.697	50.000	98.848	95
Mauritania	97.518	37.500	98.449	95
Solomon Islands	97.473	0.000	97.473	95
Cameroon	97.384	94.444	99.855	95
Cote d’Ivoire	97.272	92.188	99.787	95
Pakistan	97.182	0.000	97.182	95
Lao PDR	97.160	16.667	97.633	95
Yemen, Rep.	97.160	0.000	97.160	95
Sao Tome and Principe	97.071	75.000	99.268	95
Zambia	96.981	98.031	99.941	95
Lesotho	96.914	98.918	99.967	95
Nigeria	96.780	91.935	99.740	95
Sudan	96.646	0.000	96.646	95
Ghana	96.534	82.143	99.381	95
India	96.534	16.667	97.112	?
Vietnam	96.534	37.500	97.834	92
Nicaragua	96.310	16.667	96.925	92
Uzbekistan	96.154	0.000	96.154	92
Papua New Guinea	95.997	50.000	97.999	95
Moldova	95.371	64.286	98.347	95
Honduras	95.259	50.000	97.630	95
Bolivia	95.036	16.667	95.863	92
Bhutan	94.589	0.000	94.589	95
Philippines	94.410	0.000	94.410	?
Kiribati	94.365	0.000	94.365	95
Congo, Rep.	94.298	91.071	99.491	95
Swaziland	93.605	99.057	99.940	95
Sri Lanka	93.470	0.000	93.470	?
Morocco	93.381	0.000	93.381	?
Egypt, Arab Rep.	93.336	0.000	93.336	?
Vanuatu	93.292	0.000	93.292	95
Guatemala	93.023	64.286	97.508	95
Mongolia	92.934	0.000	92.934	92
Micronesia, Fed. Sts.	92.777	0.000	92.777	?
Samoa	92.710	0.000	92.710	95
Georgia	92.643	16.667	93.869	?
Paraguay	92.397	16.667	93.664	?
Guyana	92.375	80.769	98.534	95
Indonesia	92.352	37.500	95.220	92
Ukraine	92.174	72.222	97.826	95
El Salvador	91.972	58.333	96.655	?
Kosovo	91.950	0.000	91.950	?
Timor-Leste	91.905	0.000	91.905	95
Armenia	91.682	0.000	91.682	?
Cabo Verde	91.436	0.000	91.436	95
Albania	90.988	0.000	90.988	?
Marshall Islands	90.966	0.000	90.966	?
Fiji	90.809	0.000	90.809	?
Tunisia	90.720	0.000	90.720	?
Tonga	90.564	0.000	90.564	?
Angola	89.758	89.130	98.887	95
Macedonia, FYR	89.669	0.000	89.669	?
Jordan	89.557	0.000	89.557	?
Bosnia and Herzegovina	89.378	0.000	89.378	?
Algeria	88.775	0.000	88.775	?
Jamaica	88.529	85.294	98.313	95
Ecuador	88.439	58.333	95.183	?
Thailand	88.350	77.273	97.352	?
Serbia	88.193	0.000	88.193	?
Turkmenistan	87.903	0.000	87.903	?
Dominican Republic	87.768	64.286	95.632	95
Namibia	87.455	98.120	99.764	95
Tuvalu	87.366	0.000	87.366	95
China	87.209	0.000	87.209	?
Maldives	87.142	0.000	87.142	95
Peru	86.449	37.500	91.531	?
Iraq	86.292	0.000	86.292	?
Azerbaijan	86.091	0.000	86.091	?
St. Vincent and the Grenadines	85.689	0.000	85.689	95
Dominica	85.599	0.000	85.599	95
Belarus	85.398	37.500	90.874	?
Bulgaria	84.705	0.000	84.705	?
St. Lucia	84.593	0.000	84.593	95
Colombia	84.302	50.000	92.151	?
Grenada	83.855	0.000	83.855	95
Montenegro	83.855	0.000	83.855	?
South Africa	83.318	98.603	99.767	95
Botswana	82.894	98.913	99.814	95
Panama	80.970	64.286	93.204	95
Romania	80.859	0.000	80.859	?
Mauritius	80.836	79.167	96.008	92
Suriname	80.590	77.273	95.589	95
Costa Rica	80.277	16.667	83.564	?
Lebanon	79.450	0.000	79.450	?
Mexico	78.444	0.000	78.444	?
Kazakhstan	78.131	0.000	78.131	?
Malaysia	78.041	37.500	86.276	?
Palau	77.952	0.000	77.952	?
Gabon	77.549	93.750	98.597	95
Turkey	75.783	0.000	75.783	?
Brazil	73.994	0.000	73.994	?
Seychelles	72.764	0.000	72.764	92
Hungary	72.250	0.000	72.250	?
Venezuela, RB	72.138	58.333	88.391	?

Thus two quite different models of an international tiered pricing framework can be imagined: one under which prices are ultimately set by patent-owning pharmaceutical companies, with guidance from an internationally agreed set of non-binding principles, the other under which prices are set under WHO governance. Our intention for this paper is not to explore whether the Equitable Access Initiative is leaning more towards one model or the other. Our intention is to explore the practical feasibility of the model under which prices are set under WHO governance, using international compulsory licenses as a way to make a set of ‘equitable pricing’ principles binding.

## Discussion

Before we explain the international tiered pricing an compulsory licensing framework we have in mind, we need to unpack three concepts:

- By the right to health, we mean the entitlement of all humans to organized efforts from society that promote and improve health, and the corresponding obligations to meet those entitlements as born by governments and the international community, as enshrined in international human rights law;
- By compulsory license, we mean a legally sanctioned government action that obliges the owner of a patent to allow another company to produce and distribute a generic equivalent of the patent-protected medicine;
- By tiered pricing, we mean the practice of setting different prices for different groups of potential buyers.

### The right to health

The right to health has been enshrined in several treaties, but we focus on the International Covenant on Economic, Social and Cultural Rights (Covenant) for two reasons. Firstly, because it has been endorsed by more states than regional human rights treaties, such as the African Charter on Human and Peoples’ Rights; and secondly because it has a wider scope than treaties that confirm the right to health for specific groups, like the Convention on the Rights of the Child or the Convention on the Elimination of All Forms of Discrimination Against Women. The Covenant confirms “the right of everyone to the enjoyment of the highest attainable standard of physical and mental health” (article 12(1)) and it confirms that every state is obliged “to take steps, individually and through international assistance and co-operation, especially economic and technical, to the maximum of its available resources, with a view to achieving progressively the full realization of” the right to health and other rights recognized in the Covenant (article 2(1)). The Committee on Economic, Social and Cultural Rights that was created to monitor states’ compliance with the Covenant further clarified that states “have a core obligation to ensure the satisfaction of, at the very least, minimum essential levels of each of the rights enunciated in the Covenant, including essential primary health care”, and that these core obligations include at least ensuring the right of access to health facilities, goods and services on a non-discriminatory basis, especially for vulnerable or marginalized groups, and providing essential medicines as defined by WHO [5]. To be sure, this is about the minimum obligation, which applies to all countries, regardless of their income levels: the general legal obligation to realize the right to health progressively extends beyond essential medicines to a general duty to ensure access to all medicines that may improve people’s health. Finally, the Committee clarified that “it is particularly incumbent on States parties and other actors in a position to assist, to provide “international assistance and cooperation, especially economic and technical” which enable developing countries to fulfil their core and other obligations” [5].

### Compulsory licenses

To become or remain a member of the World Trade Organization (WTO), states have to comply with the Agreement on Trade-Related Aspects of Intellectual Property Rights (TRIPS Agreement) [6]. The TRIPS Agreement obliges WTO members to adopt certain minimum levels of intellectual property protection in their law, national or regional, including patents for new and innovative medicines. A patent gives its owner exclusive rights to the patent-protected medicine: in principle, only the patent-owner can authorize the manufacturing or importation and the distribution of the medicine that is protected by the patent, in the territory covered by the patent. This can put governments in a conflicted position: one the one hand, they have a legal obligation to ensure the provision of the medicines that people need (as discussed above); on the other hand, they may have to grant patents on new and innovative medicines, which gives the patent-owner an effective monopoly (and as a result, power over the price), unless governments use exceptional measures as we will explain below. Governments can negotiate the price of a medicine with the patent-owner, but have no guarantee that such negotiations will result in reasonable prices, especially if their home market is relatively insignificant to the patent-holding firm. However, the exclusive rights that are attached to a patent are not absolute: the TRIPS Agreement foresees several exceptions, of which we will discuss only compulsory licenses here. A compulsory license is a legally sanctioned government decision that obliges the patent-owner to provide a license to another company, allowing that other company to manufacture a generic equivalent of the medicine in question. That generic equivalent can then be distributed, under a different brand name and usually at a much cheaper price than the one set by the patent-owner. In practice, the patent-owner does not have to follow through and issue a license to a third company; the compulsory license simply signals that the patent-owner will no longer be able to oppose the manufacturing and distribution of the generic equivalent by the company appointed by the government.

Thus the ‘threat’ of a possible compulsory license often works as the ‘stick behind the door’ in price negotiations between governments and patent-owning companies. If no agreement is reached with regard to securing lower prices, governments can issue a compulsory license. This tool has been used effectively by countries that have domestic manufacturing capacity.

In countries that do not have domestic manufacturing capacity, however, compulsory licenses have proven less effective with regard to securing lower prices. This is due to the fact that the reach of a compulsory license is limited to the territory of the government that issues it. The government of a given country could, in theory, issue a compulsory license for a given medicine to a manufacturer based in another country, but that license has no legal validity in that other country. So if the given medicine is protected by a patent in that other country, the manufacturer would still not be allowed to produce the generic equivalent, even if it is for export purposes only. A second compulsory license, issued by the government of the country where the manufacturer is located, would be required. And that is difficult because of TRIPS Agreement requirement that compulsory licensing be “predominantly for the supply of the domestic market” [6].

The public outrage created by some excessively stringent interpretations of the TRIPS Agreement forced the WTO to adopt, in Doha in 2001, a Declaration on the TRIPS Agreement and Public Health, which confirmed that “the TRIPS Agreement does not and should not prevent Members from taking measures to protect public health”, and that every state “has the right to grant compulsory licenses and the freedom to determine the grounds upon which such licenses are granted” [7]. The Doha Declaration also called for a solution to the problem explained above: the requirement that compulsory licensing be “predominantly for the supply of the domestic market,” which particularly affects least developed countries without local manufacturing capacity. As a result, the WTO General Council released a decision in August 2003, waiving the ‘for domestic purposes only’ requirement, thus permitting developing countries to import generic medicines produced under compulsory licenses issued for export purposes [8]. This decision was formalized in December 2005 and will – if ratified by a sufficient number of WTO members – create a special compulsory license system in which an importing country would notify the WTO of its intention to use the system, while the exporting country would grant a compulsory license to export pharmaceuticals to the country in question [9]. Until then – the deadline for ratification has been extended until December 2015 – the August 2003 waiver remains valid.

So far, this special compulsory license regime in the making has only been used once in 2007, when a Canadian generic manufacturer obtained permission to export antiretroviral medicines to Rwanda. The manufacturer made only two shipments of drugs to Rwanda over a six year period and declined to renew the license because of the complexity, cost and limited duration of the license [10]. Legislative efforts to streamline the licensing process were voted down twice in Canadian parliament, at least in part because of pharmaceutical industry opposition [11]. Furthermore, this special compulsory license regime limits the competition between potential generic manufacturers to those based in countries that issue compulsory licenses, and thus has only limited effect on market prices.

### Tiered pricing

In essence, tiered pricing indicates the practice of setting different prices for different groups – tiers – of potential buyers or national markets. As an illustration of this practice, McDonalds sets different prices for hamburgers sold in New York and in Johannesburg. In theory, tiered pricing is completely unrelated to patent protection and compulsory licenses. When a company applies for a patent, the outcome of that application does not depend on whether or not the company commits to tiered pricing. And when a government is in a position to issue a compulsory license, the commitment of the patent-owner to tiered pricing – or the absence thereof – does not affect the right of the government to issue a compulsory license. Yet when tiered pricing is discussed in the context of medicines, it is usually presented as an alternative for, or a correction to, the present intellectual property regime for medicines. The idea is that tiered pricing in accordance with a widely endorsed set of principles would provide a middle ground between prices arbitrarily set by patent-owning companies, and compulsory licenses arbitrarily issued by governments [12].

### An international tiered pricing and compulsory licensing framework

For compulsory licenses to have the same ‘stick behind the door effect’ on price negotiations in countries without domestic manufacturing capacity – as they have in countries with domestic manufacturing capacity – they should have an international reach. We suggest that an international tiered pricing and compulsory licensing framework could be created through a convention in accordance with article 19 of the WHO constitution. According to article 19, the WHA “shall have authority to adopt conventions or agreements with respect to any matter within the competence of the Organization”, and “[a] two-thirds vote of the Health Assembly shall be required for the adoption of such conventions or agreements, which shall come into force for each Member when accepted by it in accordance with its constitutional processes” [13]. At present, a two thirds vote means 130 members out of 194 members. It is possible therefore that a WHO Convention on International Tiered Pricing and Compulsory Licensing (Convention) could lay out the principles needed to calculate ‘reasonable’ prices for patent-protected medicines. This Convention would not have the power to impose these prices upon patent-owning companies directly, but it could do so indirectly, by promoting and facilitating ‘international compulsory licensing’ whenever a patent-owner charges prices above the reasonable level. The Convention as we imagine it would appoint a panel that could issue international compulsory licenses upon the request of governments that were unable to negotiate reasonable prices with the patent-owning companies. This would go a long way to solving the problems faced in the Canada and Rwanda example detailed above. Once issued, these compulsory licenses would be considered valid in all counties that have ratified the Convention, which would allow all companies with manufacturing capacity for the medicine in question to submit a bid to the government that successfully requested the international compulsory license – thus creating competition between all companies with manufacturing capacity for the medicine in question that are based in one of the countries that ratified the Convention. Once the purchasing country made its choice, the government of the country where the manufacturer is based would issue a final compulsory license, to satisfy the WTO requirement that the exporting country grant a compulsory license to export pharmaceuticals (but that would be a mere formality).

To make our suggestion less abstract, we considered a very rudimentary set of principles, based on two criteria often used in tiered pricing debates: GNI per capita and burden of disease [12]. (Obviously, more sophisticated set of principles could and probably should be developed and included in the Convention.) We then applied these principles to one particular medicine: Atripla, which is considered as the ideal first line antiretroviral therapy for developing countries, but still not used as such in many countries because of the price [14]. Based on its desirability and pricing, we can assume that the Global Fund will make access to this medicine a priority for the Equitable Access Initiative. Furthermore, since Atripla is a medicine for people living with AIDS, and reliable HIV/AIDS prevalence data are easy to find, the drug is a good candidate for our exercise – which is merely an illustration, not a comprehensive analysis. According to the 2013 ‘Untangling the Web of Antiretroviral Price Reductions’ report of Médecins Sans Frontières, Merck sells Atripla at US$613 per person per year to several ‘Category 1’ developing countries, and at $1,003 per person per year to a few ‘Category 2’ developing countries [15]. The cheapest generic equivalent costs $158 per person per year. Which of these prices is the most ‘reasonable’?

To answer the question, we first looked up the price for Altripla in Belgium: €822.17 for 30 days [16], which makes €10,003 per year, or $12,530 per year. Thus in comparison to Belgium, ‘Category 2’ countries receive a 92% discount, while ‘Category 1’ countries receive a 95% discount. The cheapest generic equivalent results in a 99% ‘discount’.

Then we considered that all developing countries should receive a first discount based on their Gross National Income (GNI) per capita, because the GNI per capita provides an approximation of potential government revenue, and therefore of potential government ability to purchase and distribute or subsidize medicines. For example, if the GNI per capita of Brazil is $11,630, while the GNI per capita of Belgium is $44,720, according to World Bank data [17], the ability of the Government of Brazil to purchase or subsidize Atripla (and other medicines) is considered to be at 26% of the ability of the Government of Belgium, and we assumed that Brazil ought to receive a first discount of 74%. (According to the 2013 ‘Untangling the Web of Antiretroviral Price Reductions’ report of Médecins Sans Frontières, the Government of Brazil receives no systematic discount: it is neither a ‘Category 1’ nor a ‘Category 2’ country for Merck.)

Then we considered that all developing countries should receive a second discount in accordance with the prevalence of the disease for which the medicine is needed – in this case, adult HIV prevalence. The justification for this second discount is again based on countries’ ability to pay for medicines: at equal potential government revenue, a country that has to provide AIDS treatment to 10 out of 100 inhabitants can afford to pay – per patient – only 10% of what a country that has to provide AIDS treatment to 1 out of 100 inhabitants can pay. The first country therefore ought to receive an additional 90% discount. Again we looked at World Bank data [17], except for the reference country (Belgium) because the World Bank database does not include an HIV prevalence estimate for Belgium. According to the epidemiological factsheet generated by the UNAIDS website, the adult HIV prevalence in Belgium is 0.25% [18]. For countries for which the World Bank database does not include an estimate of adult HIV prevalence, we assumed – for the sake of this exercise – that HIV prevalence is not significantly high enough to warrant an additional discount.

According to our estimates, represented in Table 1, only 13 out of the 127 developing countries we included receive a ‘reasonable’ discount from Merck: a discount that results in a financial burden that does not exceed the financial burden on the Government of Belgium if we take GNI per capita and adult HIV prevalence into account. These countries are: Bhutan, Kiribati, Vanuatu, Samoa, Timor-Leste, Cabo Verde, Tuvalu, Maldives, St Vincent and the Grenadines, Dominica, St Lucia, Grenada, Panama, and The Seychelles. Under the Convention proposed here, they could still issue a (domestic) compulsory license but would not obtain an international compulsory license.

The other 114 countries could, under the Convention proposed here, apply for an international compulsory license. A panel of experts under WHO governance would verify GNI and HIV prevalence data, and perhaps give Merck a final chance to adjust its tiered pricing in accordance with the principles of the Convention. If that were unsuccessful the panel would issue an international compulsory license.

If an international compulsory license would result in a 99% ‘discount’, even that would not be sufficient for 39 countries: they would need additional assistance – from the Global Fund, for example.

Most of the other 75 countries (out of the 114 countries for which the present Merck discount is insufficient) could pay the price of the cheapest generic equivalent without facing a higher relative financial burden than the Government of Belgium; some could even pay up to four times the cost of the cheapest generic equivalent without facing a higher relative financial burden than the Government of Belgium. Mauritius, for example, would according to our estimates be able to pay 4% of the price for Belgium but receives only a 92% discount (instead of a 96% discount). If the Convention panel would issue an international compulsory license for Mauritius (or another country in a similar situation), it could stipulate that some royalties would have to be paid.

So far, our proposal was based on the assumption that the price for Belgium is indeed reasonable, and can be used to calculate reasonable tiered pricing for developing countries, which may not be the case for all new medicines [19]. The Convention could also allow the panel to use, at times, a “cost-based yardstick approach” [20]: demanding information about the real cost of a medicine, setting a reasonable reference price for high income countries, then calculating reasonable prices for middle and low income countries accordingly. Thus even high income countries could benefit from this Convention, which may increase the likelihood of obtaining 130 votes in favor at the WHA.

Finally, we would argue, in line with Elliott, that “facilitating compulsory licensing of pharmaceuticals” is, in the given circumstances, a matter of legal obligation under article 2(1): states are indeed obliged “to take steps, individually and through international assistance and co-operation, especially economic and technical”, to realize the right to health, and facilitating compulsory licensing for countries that needed it is but one of these steps [21].

## Summary

In summary, we would argue that Berkley’s assumption, according to which “it is for manufacturers to set the prices of vaccines [and medicines]” [1], is made too hastily. This assumption is probably based on thinking that no technical solution can be found within the present scope of the international intellectual property regime that could force patent-owners to accept guidelines set by international organizations. But as we demonstrated here, there is a solution, and a relatively simple one too. What it takes is the political will of WHO and 130 of its member states. Whether this political will exists is food for a different debate.

## Abbreviations

GNI: Gross National Income
TRIPS Agreement: Agreement on trade-related aspects of intellectual property rights
WTO: World trade organization
